# Effect of temperature and humidity on mechanical properties and constitutive modeling of pressure-sensitive adhesives

**DOI:** 10.1038/s41598-024-64960-2

**Published:** 2024-06-25

**Authors:** Weiquan Luo, Wenzhen Chen, Dashun Liu, Xiaofeng Huang, Baoguang Ma

**Affiliations:** Center for Engineering Materials and Reliability, Guangzhou HKUST Fok Ying Tung Research Institute, Guangzhou, 511458 China

**Keywords:** Mechanical engineering, Materials science, Soft materials

## Abstract

Pressure-sensitive adhesives (PSAs) are crucial for the structural and functional integrity of flexible displays. Investigating the intricate mechanical properties of PSAs can help enhance product quality and performance. This study conducts systematic mechanical tests, including uniaxial tensile, compression, planar shear, and stress relaxation, on PSAs at temperatures ranging from – 25 to 85 ℃ and relative humidity levels from 0 to 90%. Our findings reveal that the Anssari-Benam model accurately describes the hyperelastic behavior of PSA materials under large deformation, outperforming the Ogden model by requiring fewer parameters and better preserving convexity. Moreover the results show that temperature markedly affects PSA properties, particularly near the glass transition temperature (Tg), with lower temperatures leading to decreased elasticity and higher temperatures aiding in stress relaxation. Similarly, humidity impacts PSA behavior, increasing elasticity and decreasing stiffness, especially noticeable in stress relaxation tests. These findings highlight the substantial influence of environmental conditions on the material properties of PSAs and underscore the necessity of understanding both hyperelastic and viscoelastic responses for their application in flexible technologies. This research provides critical insights for the optimal utilization of PSAs in the rapidly evolving field of flexible electronics, including OLED displays.

## Introduction

Flexible organic light-emitting diode (OLED) displays incorporate a complex layering of components, such as the cover window, circular polarizer, touch sensor, OLED panel, and flexible substrate, which are cohesively bonded using pressure-sensitive adhesives (PSAs). These adhesives are integral to the structural integrity and functionality of OLED displays^1–3^. PSAs are characterized by their ability to adhere rapidly to a variety of surfaces, a property that is crucial in connecting components in a display. The efficacy of PSAs in adhering to various surfaces can be attributed to their specialized polymer composition, which optimally integrates adhesive performance with material properties that are adaptable to a wide range of application environments^4^. In addition to their adhesive properties, PSAs serve an important role as stress-dissipating layers, thereby enhancing the structural resilience of devices under conditions of flexural stress^5^. This enhancement is crucial in applications where flexibility and mechanical stability are paramount. The mechanical behavior of PSAs, however, is profoundly influenced by external factors, including environmental conditions such as temperature and humidity, as well as various stress modes^6,7^. Therefore, understanding the mechanical responses of PSAs to these factors is critical. This knowledge is indispensable for developing a comprehensive constitutive model for PSAs, designed to predict and optimize their performance under diverse real-world conditions.

In the realm of mechanical performance studies of PSAs, various investigations have provided insights into their behavior under different environmental and loading conditions. Chiang et al. utilized rheological tests to evaluate the influence of frequency and temperature on the viscosity of PSAs, uncovering a pronounced temperature sensitivity^8^. Their findings indicated a sharp decline in viscoelastic properties when transitioning from high to low temperatures, and they noted that PSAs with higher elastic moduli do not consistently exhibit increased tack across varying temperature conditions and phase angles. Similarly, focusing on temperature effects, Zhao et al. conducted experiments involving uniaxial tensile and planar shear on PSA films, analyzing the impact of strain rate on mechanical behavior by varying the loading rate^9^. Their results showed that, at the same strain level, both uniaxial tensile and shear stresses in PSA films increased with higher strain rates, underscoring the importance of strain rate considerations. Regarding the role of PSAs in stress buffering, Ha et al. compared flexible display bending test results with simulation outcomes and found that PSAs could function as a buffering layer, reducing stress on both surfaces^10^. However, their model treated the PSA layer as linearly elastic, which limits its applicability in nonlinear, large deformation conditions. Building on this, Jia et al. developed a hyperelastic constitutive model specifically for optically transparent adhesives, based on uniaxial tension and simple shear experiments^11^. Although this model achieved high fitting accuracy, it did not account for the rate-dependent behavior of viscoelastic materials, highlighting a gap in current modeling approaches.

In the current study, a systematic investigation is conducted to examine the viscoelastic and hyperelastic mechanical behavior of PSAs under varied environmental and loading conditions. This research involves a series of rigorously designed experiments to characterize the viscoelastic and hyperelastic responses of PSAs to different temperature and humidity levels, as well as stress modes such as uniaxial tension, compression, and planar shear^12^. These experimental setups facilitate the determination of both hyperelastic and viscoelastic parameters for PSAs. Additionally, the study investigates the effects of environmental factors, specifically temperature and humidity, and initial strains on the mechanical behavior of PSAs. The findings from this research are critical, not only in elucidating the performance characteristics of PSAs under complex operational conditions but also in significantly enhancing the reliability and longevity of flexible OLED displays in practical applications.

## Theory

PSAs, due to their high-molecular-weight polymer composition, inherently exhibit a combination of hyperelastic and viscoelastic behaviors. These mechanical properties are notably influenced by environmental factors, such as temperature and moisture, which are critical for flexible displays^13^. Flexible displays undergo constant cyclic deformations and dynamic transformations during operation, and are subjected to temperature fluctuations^14^. As the bonding agents for all film layers in display modules, the PSA requires high adhesion properties to resist the dynamic force. Otherwise, the PSA may detach from the glass and cause air bubbles and wrinkles on the surface, which impair the visual effects.

Therefore, developing a constitutive model that can accurately describe the hyperelastic and viscoelastic behaviors of PSAs is critically important. These models can help to improve the accuracy of device design and analysis results when predicting the performance and longevity of flexible displays using finite element simulation technology^10^.

### Hyperelastic models

PSAs, primarily composed of high molecular weight polymers, are capable of undergoing substantial deformations and demonstrate nonlinear stress–strain relationships. Over recent decades, two principal approaches have been developed to model their mechanical behavior: micromechanical network models and hyperelastic potential models, also known as phenomenological models^9^. Micromechanical network models provide a statistical interpretation of the molecular chains in the elastomer, allowing for the simulation of complex material behaviors with a relatively small number of physically meaningful parameters. However, their integration into finite element analysis often requires significantly more computational resources compared to phenomenological models. In contrast, phenomenological models utilize a continuum mechanics approach, focusing on the macroscopic behavior of the material rather than its microscopic structure. These models, crucial in describing nonlinear elastic behavior, formulate the strain energy density as a function of the deformation state. The elastic potential in these models is typically expressed through the strain invariants or the principal stretches^15^. In this work, several models, including Neo-Hookean, Mooney-Rivlin, Yeoh, Ogden, and Anssari-Benam models, were used to characterize the hyperelastic properties of the OCA material^16–19^.

Neo-Hookean Model:

1 $$U = C_{10} (\overline{I}_{1} - 3)$$

Mooney-Rivlin Model:

2 $$U = C_{10} (\overline{I}_{1} - 3) + C_{01} (\overline{I}_{2} - 3)$$

Yeoh Model:

3 $$U = \sum\limits_{i = 1}^{3} {C_{i0} (\overline{I}_{1} - 3)^{i} }$$

Ogden Model:

4 $$U = \sum\limits_{i = 1}^{N} {\frac{{2\mu_{i} }}{{\alpha_{i}^{2} }}} (\lambda_{1}^{{\alpha_{i} }} + \lambda_{2}^{{\alpha_{i} }} + \lambda_{3}^{{\alpha_{i} }} - 3)$$

Anssari-Benam Model:

5 $$U = \frac{{3\left( {n - 1} \right)}}{2n}\mu N\left[ {\frac{1}{{3N\left( {n - 1} \right)}}(\lambda_{1}^{\alpha } + \lambda_{2}^{\alpha } + \lambda_{3}^{\alpha } - 3) - \ln \left( {\frac{{\lambda_{1}^{\alpha } + \lambda_{2}^{\alpha } + \lambda_{3}^{\alpha } - 3N}}{3 - 3N}} \right)} \right]$$

In the above models, *U* represents the strain energy density function for incompressible material, such as PSAs, where *C*, *μ*, *n, N*, and *α* are material parameters. *I*_*1*_ and *I*_*2*_ denote the first and second invariants of the deviatoric strain tensor.

Several material characterization tests are needed to generate data for the calibration of the constitutive models for PSAs. For calibrating the hyperelastic material models, one or more of the tests listed below needs to be performed on the PSAs: uniaxial tensile, simple shear, biaxial tensile, compression and/or bulk compression tests.

### Viscoelastic models

PSAs are distinctively characterized by their viscoelastic properties, alongside non-linear elastic behavior, influenced by their material composition. These properties, particularly stress relaxation and creep, require a specialized viscoelastic constitutive model for accurate representation. To achieve this, models incorporate basic elements like Hooke’s spring and the basic dashpot to represent ideal elastic and viscous behaviors, respectively. This approach allows for capturing the complex viscoelastic behaviors of PSAs^20^. In practice, models such as Maxwell, Kelvin, three-parameter, and generalized Maxwell are commonly used for this purpose^1^. The Prony series is particularly effective for modeling PSAs’ stress relaxation function, crucial in determining their viscoelastic parameters accurately.

6 $$\tau (t) = G_{0} \int\limits_{0}^{t} {g_{R} (t - s)} \dot{\gamma }(s)ds$$

7 $$P(t) = - K_{0} \int\limits_{0}^{t} {k_{R} (t - s)} \dot{\varepsilon }^{vol} (s)ds$$

In the proposed equation, *τ* represents the shear stress, and *P* denotes the hydrostatic pressure; these terms are critical in characterizing the relaxation stress changes in PSAs. Specifically,* τ* accounts for the changes due to shear deformation, while *P* relates to those arising from volumetric deformation. Further, *G*_0_ and *K*_0_ represent the instantaneous shear modulus and the instantaneous elastic bulk modulus, respectively. The functions *g*_*R*_(*t*) and *k*_*R*_(*t*) denote the time-dependent, dimensionless forms of the shear and volumetric relaxation moduli. These moduli, critical for describing the time-dependent viscoelastic behavior of PSAs, are expressed through the Prony series.

8 $$g_{R} (t) = 1 - \sum\limits_{i = 1}^{N} {g_{i} \left( {1 - e^{{ - \frac{t}{{\tau_{i} }}}} } \right)}$$

9 $$k_{R} (t) = 1 - \sum\limits_{i = 1}^{N} {k_{i} \left( {1 - e^{{ - \frac{t}{{\tau_{i} }}}} } \right)}$$

where *τ*_*i*_ represents the state variable governing stress relaxation, *g*_*i*_ and *k*_*i*_ stand for the shear relaxation coefficient and volumetric relaxation coefficient, respectively, *N* denotes the number of terms in the Prony series.

For the calibration of the viscoelastic model of PSAs, one effective method involves conducting dynamic mechanical analysis tests. These tests generate master curves that depict the storage and loss moduli of the PSAs, crucial for calibrating the Prony series^18^. An alternative method is to use relaxation or creep test data, obtained under various strain rates and temperatures, for Prony series calibration. In this study, stress relaxation tests were specifically conducted to assess the viscoelastic properties of PSAs and to obtain the necessary parameters for the constitutive model.

## Experiment

To investigate the hyperelastic and viscoelastic properties of PSAs, this study conducted extensive mechanical experiments on representative samples of CEF3501, a PSA provided by 3M Company. These experiments included uniaxial tensile, compression, and planar shear tests, primarily aimed at determining the parameters critical for developing the hyperelastic constitutive model. Additionally, a series of stress relaxation experiments were conducted to ascertain the viscoelastic parameters for PSAs.

### Sample size evaluation and preparation

**Figure 1:**
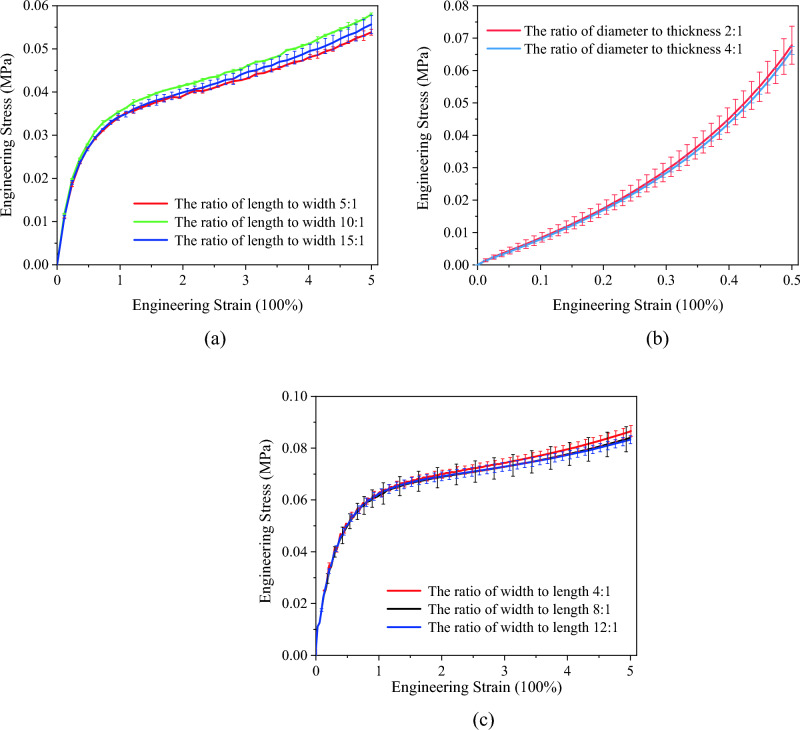
Stress–strain curves of PSA specimens with different length–width ratios: (**a**) uniaxial tensile test; (**b**) uniaxial compression test; (**c**) planar shear test.

Due to the absence of uniform standards for preparing PSA material samples, initial experiments were conducted to assess the impact of sample size, focusing on varying length-to-width ratios, on their mechanical properties. Specimen dimensions for both uniaxial tensile, uniaxial compression and planar shear tests are detailed in Tables 1, 2 and 3. Tests were performed using specimens with different length–width ratios, under a constant tensile rate at a controlled temperature of 25 °C. Stress–strain data derived from these tests are presented in Fig. 1a–c.

**Table 1:** The parameters of uniaxial tensile test specimens.

No.	Length (mm)	Width (mm)	Thickness (mm)	Length to width ratio
1	50	10	0.2	5:1
2	100	10	0.2	10:1
3	150	10	0.2	15:1

**Table 2:** The parameters of uniaxial compression test specimens.

No.	Diameter (mm)	Thickness (mm)	Diameter to thickness ratio
1	5	2.5	2:1
2	10	2.5	4:1

**Table 3:** The parameters of planar shear test specimens.

No.	Length (mm)	Width (mm)	Thickness (mm)	Width to length ratio
1	10	40	0.2	4:1
2	10	80	0.2	8:1
3	10	120	0.2	12:1

At a constant temperature of 25 °C, trends in the mechanical properties for samples with different length–width ratios showed consistent patterns. Figure 1 shows the observed variations in sample size resulted in deviations within a 10% margin, suggesting that the material's properties are relatively unaffected by size differences. To ensure compatibility with the testing equipment, sample dimensions were standardized to 50 mm × 10 mm × 0.2 mm for uniaxial tensile tests and 10 mm × 80 mm × 0.2 mm for planar shear tests. The sample dimension for uniaxial compression test was 10 mm × 2.5 mm (Diameter × Thickness).

## Experimental procedure

- Hyperelastic Experiment

**Figure 2:**
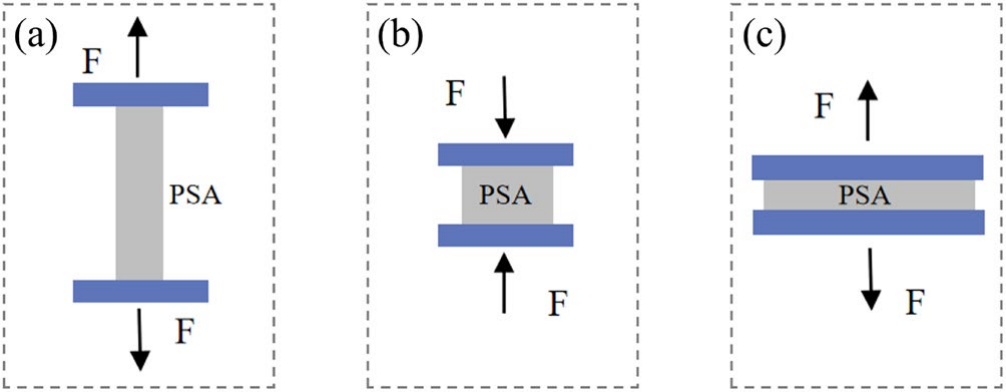
Schematic diagrams of the hyperelastic tests: (**a**) uniaxial tensile; (**b**) compression; (**c**) planar shear.

In this study, a series of uniaxial tensile, compression, and planar shear tests were performed to examine the hyperelastic mechanical behavior of the PSAs. Schematic diagrams, which illustrate the setups for each of these tests, are provided in Fig. 2 for reference.

**Figure 3:**
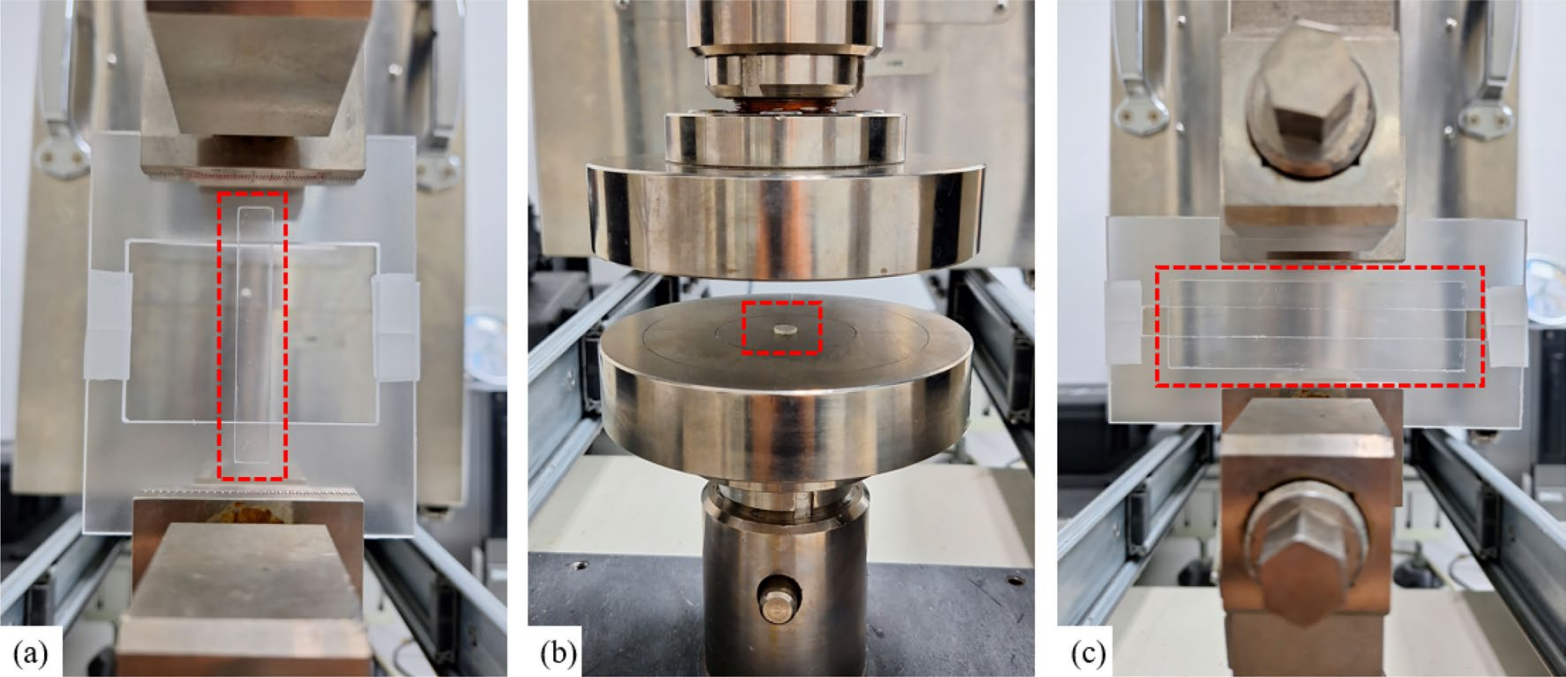
The testing machine with samples: (**a**) uniaxial tensile; (**b**) compression; (**c**) planar shear.

To investigate the effects of temperature and humidity on the hyperelastic mechanical behaviors of PSAs, all experiments were carried out at a constant loading rate $\dot{\varepsilon }$ = 0.01 s^−1^ on a universal testing machine (Instron 34TM30), which was equipped with a precision-controlled temperature and humidity chamber. The testing machine and the samples are shown in Fig. 3, along with a board frame that prevents mechanical damage from the clamps. The specific temperature conditions during the experiments, outlined in Table 4, cover the temperature ranges that flexible displays containing PSAs might encounter during operation or storage. The condition of 90% relative humidity at 55 °C was chosen to test PSA performance in high-moisture environments.

**Table 4:** Environmental conditions for hyperelastic tests.

T (℃)	RH (%)	μ [MPa]	α [−]	n [−]	N [−]	R^2^
− 25	0	0.651	0.155	22.814	1.032	0.978
25	0	0.143	0.521	47.112	1.628	0.985
55	0	0.044	1.078	1.325	3.798	0.998
55	90	0.023	1.359	1.471	7.934	0.997
85	0	0.057	0.872	2.162	2.857	0.989

In the uniaxial tensile and planar shear tests, the strain applied to the PSA samples was extended to 500%. These tests were specifically designed to evaluate the hyperelastic behavior of the PSAs under different loading modes. In contrast, for the uniaxial compression experiments, the strain was limited to 50%. Special measures were implemented to ensure the samples could expand unconfined during compression. This involved placing thin, rigid sheets with a thickness of 0.5 mm on the surfaces of both clamps, and meticulously lubricating them to minimize potential friction between the samples and fixtures. Upon completion of all experiments, the displacement-load data collected were transformed into stress–strain curves through post-processing for detailed analysis.

- Viscoelastic Experiment

In this study, stress relaxation tests were performed on the same machine to assess the viscoelastic characteristics of PSAs. To investigate the viscoelastic properties, uniaxial stress relaxation tests were conducted under controlled environmental conditions, as outlined in Table 4, using three specimens in each experimental group. The PSA samples were loaded up to 50%, 100%, and 200% engineering strain, and then were held steady for 900 s. The time-dependent changes in stress were recorded and subsequently utilized to determine the parameters for the viscoelastic constitutive model.

## Results and discussion

### Hyperelastic behavior of PSAs

Impact of Temperature

**Figure 4:**
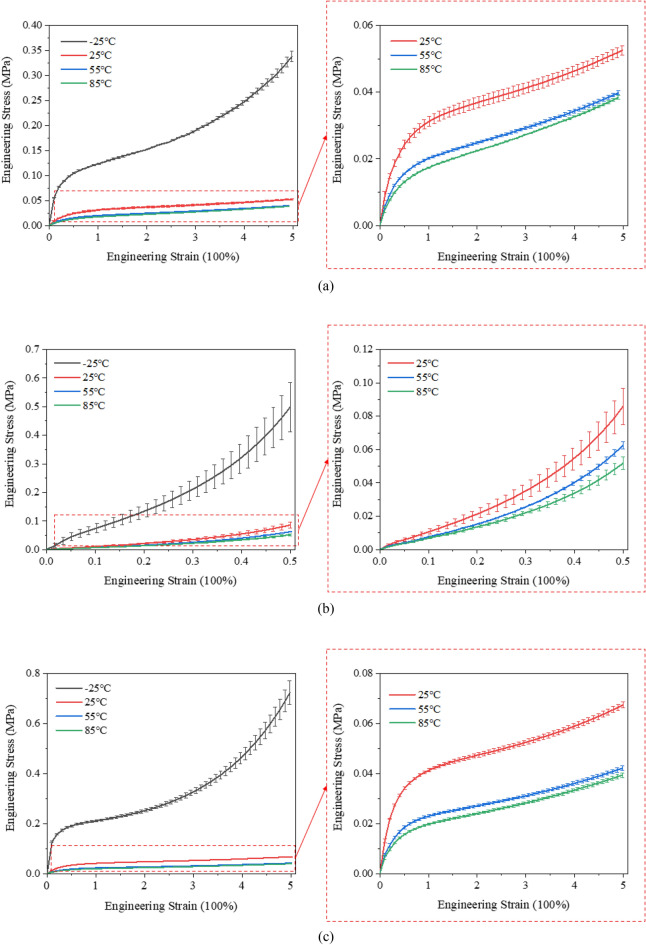
Experimental stress-strain curves for PSAs at different temperatures (− 25 ℃, 25 ℃, 55 ℃ and 85 ℃): (**a**) uniaxial tensile; (**b**) compression; (**c**) planar shear.

Under varying temperature conditions (− 25 °C, 25 °C, 55 °C, and 85 °C), PSAs were subjected to uniaxial tensile, compression, and planar shear tests. The resultant stress–strain curves from these experiments are illustrated in Fig. 4.

The engineering stress–strain curves of the PSAs being investigated distinctly exhibit S-shaped nonlinear characteristics, demonstrating strain hardening effects. These curves indicate three distinct deformation stages during the testing process. Initially, the stress–strain relationship is approximately linear, defining the linear-elastic stage where stress is directly proportional to strain. As stretching continues, a second region emerges, characterized by a gentle slope in the stress–strain curve, which is indicative of a highly elastic stage. Subsequently, the PSAs exhibit increased resistance to deformation, indicating a pronounced strain hardening effect.

The stress–strain curve at − 25 °C, as illustrated in Fig. 4, shows marked differences compared to the curves at 25 °C, 55 °C, and 85 °C. This variation can be explained by considering the glass transition temperature (T_*g*_) of the PSA material, typically between − 80 °C and 0 °C^21^. At − 25 °C, near or within the T_*g*_ range, PSAs transition from a rubbery to a glassy state, resulting in reduced polymer chain mobility and a stiffer material less capable of large deformations. This shift impacts the stress–strain behavior, leading to a markedly different curve at lower temperatures.

Conversely, at temperatures like 25 °C, 55 °C, and 85 °C, which are above the PSA’s T_*g*_, the material remains in a rubbery state with greater polymer chain mobility. This results in stress–strain curves characteristic of highly elastic materials, showing significant strain without corresponding increases in stress.

Furthermore, the stress–strain curves at 55 °C and 85 °C are observed to be relatively similar, indicating that the PSA material maintains a highly elastic state within this temperature range, with minimal variation in its hyperelastic properties. This similarity can be primarily attributed to the low T_*g*_ of PSAs, typically ranging between − 80 and 0 °C. Consequently, at both 55 °C and 85 °C, the material remains well above its T_*g*_, ensuring it is in a rubbery and highly elastic state. At temperatures significantly above the T_*g*_, the PSAs exhibit increased chain mobility and flexibility, leading to pronounced elastic behavior.

In the temperature range of 55–85 °C, the molecular structure of the PSA facilitates extensive elastic deformation with nearly consistent hyperelastic parameters, suggesting a threshold beyond which further temperature increases do not significantly alter the material’s hyperelastic response. This behavior indicates a consistent resistance to deformation despite the temperature increase and underscores the resilience of PSAs in maintaining their mechanical integrity under varying thermal conditions. Such a finding is crucial for applications where PSAs are expected to perform reliably in higher temperature environments. This resilience, coupled with the observed consistency in hyperelastic parameters, as highlighted in Fig. 4, provides valuable insights into the thermal stability and performance consistency of PSAs across different operating environments.

- Impact of humidity

PSAs are frequently assessed for their reliability and durability in environments characterized by high temperatures and humidity, such as conditions of 55 °C and 90% RH. The behavior of PSAs under such elevated thermal and moisture levels plays a pivotal role in determining the reliability and durability of flexible devices that incorporate multiple layers of these adhesives. In high-humidity conditions, PSAs are prone to moisture absorption, often reaching a state of supersaturation at elevated temperatures^22^. This phenomenon can result in bulk plasticization within the PSA material, thereby altering its hyperelastic and viscoelastic properties. Hence, a thorough characterization of PSA properties under these hygrothermal conditions is crucial to ensure their effective performance and longevity in practical applications.

**Figure 5:**
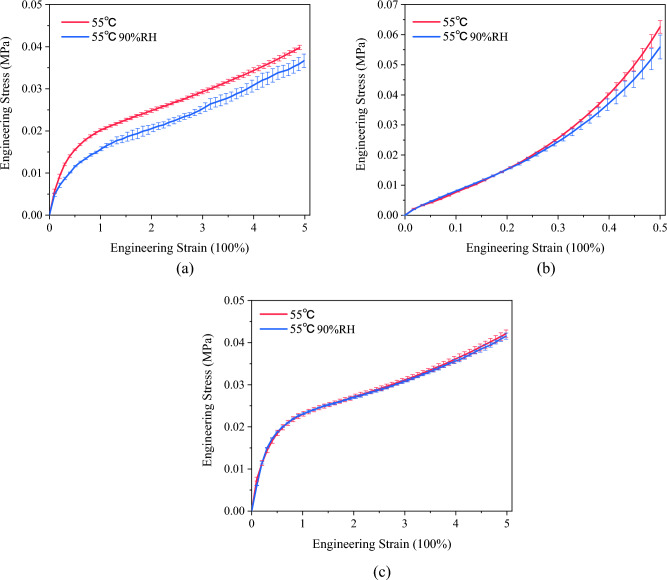
Experimental stress-strain curves for PSAs under different humidity conditions (55 ℃ and 55 ℃ 90% RH): (**a**) uniaxial tensile; (**b**) compression; (**c**) planar shear.

To investigate the impact of humidity on the hyperelastic mechanical behavior of PSAs, this study conducted experiments at a constant temperature of 55 °C under two distinct relative humidity conditions: 0% RH and 90% RH. Similar to the aforementioned experiments, the PSAs were subjected to uniaxial tensile, compression, and planar shear tests. The stress–strain curves obtained from these tests, which offer an extensive depiction of the material's response under varying humidity conditions, are comprehensively illustrated in Fig. 5.

The results presented in Fig. 5 clearly indicate that humidity differentially affects the mechanical behavior of PSAs in uniaxial tensile, compression, and planar shear tests. There is a notable variance in the material's response to each testing modality when subjected to high humidity conditions. Generally, moisture absorption in adhesives occurs through free water, causing plasticization, and bound water, which forms hydrogen bonds leading to swelling and a reduced bulk modulus and T_*g*_^23^. At 90% RH, moisture acts as a plasticizer in the PSA matrix, enhancing polymer chain mobility, reducing intermolecular forces, and allowing for increased elasticity and viscosity, thereby softening the material.

The results in Fig. 5 reveal that humidity distinctively influences the mechanical behavior of PSAs under uniaxial tensile, compression, and planar shear tests. At 90% RH, the material exhibits notable changes: it becomes more compliant and ductile in uniaxial tensile tests, showing higher strains at lower stress levels. In compression tests, differences in response are less pronounced at small strains but become more evident at higher strains, with a noticeable reduction in stiffness. However, planar shear tests demonstrate minimal variation, suggesting consistent shear properties across different humidity levels. These behavioral variations can be attributed to humidity-induced changes in the PSA’s microstructure and molecular interactions as follows:

Firstly, humidity can impact the orientation of polymer chains. In uniaxial tensile tests, where the stress direction aligns with elongation, humidity may facilitate polymer chains to align more in this direction, enhancing the material's extensibility. In contrast, in compression tests, humidity may lead to greater alignment of polymer chains along the compression direction, modifying the material's compressive resistance^22^.

Secondly, humidity plays a role in altering intermolecular interactions within the PSA. The presence of moisture can change the way molecules interact, such as through the formation or breaking of hydrogen bonds. These alterations in molecular interactions significantly affect the material's elasticity and viscoelastic properties. Under different stress conditions, the effects of humidity on these molecular dynamics can lead to distinct changes in the material's mechanical behavior. While it enhances extensibility in uniaxial tensile tests, in compression tests, it influences the compressive behavior, and in planar shear tests, the impact is minimal, maintaining consistent shear properties^24^.

- Constitutive model investigation

**Figure 6:**
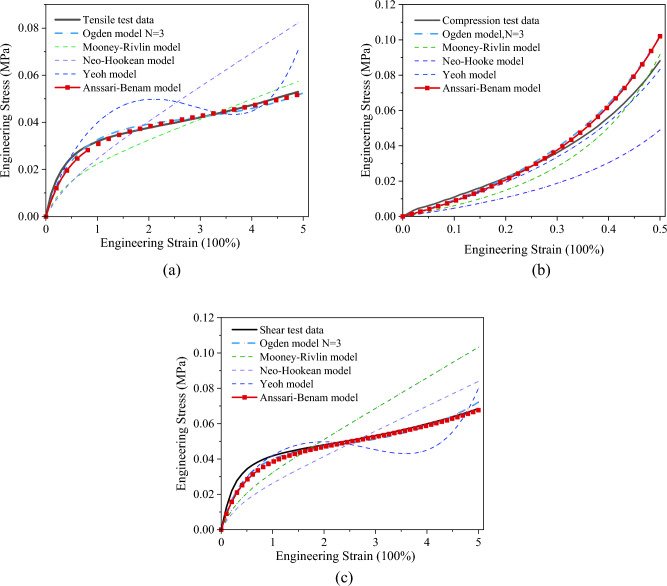
Fitting results of analytical expressions and experimental data. The analytical expressions encompass the third-order Ogden, Mooney-Rivlin, Neo-Hookean, Yeoh, and Anssari-Benam models. Mechanical characterizations involve (**a**) uniaxial tensile, (**b**) compression, and (**c**) planar shear tests.

Based on the results of the uniaxial tensile, compression, and planar shear tests under the various temperature and humidity conditions described above, this section investigates the appropriate constitutive model to describe their hyperelastic mechanical behavior. The focus is on comparing the accuracy and applicability of five widely employed models: Neo-Hookean, Mooney-Rivlin, Yeoh, Ogden, and Anssari-Benam. The material parameters are determined through minimizing the objective function *E*, where $E = \sum\limits_{i} {\left( {f_{i}^{model} \left( {\left. {x_{i}^{test} } \right|a} \right) - y_{i}^{test} } \right)^{2} }$. $y_{i}^{test}$ and $x_{i}^{test}$ are experimental stress and strain. $f_{i}^{model} \left( {\left. {x_{i}^{test} } \right|a} \right)$ is the stress calculated from the strain energy density function, where a is the material parameters. By optimizing this function using the genetic algorithm and the least squares method, parameters for the model were determined. Figure 6 depicts the fitting curves for these five models, derived under controlled conditions at 25 °C and 0% RH.

**Figure 7:**
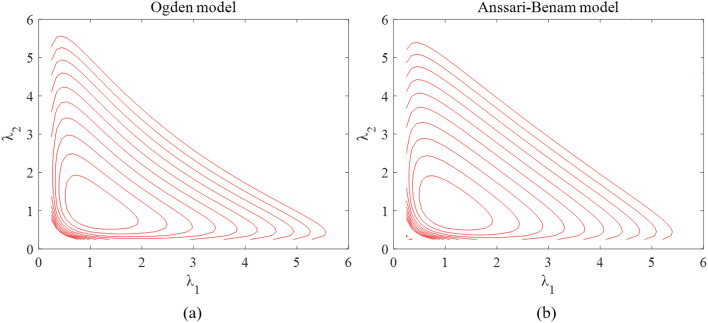
Iso-energy plots in the principal stretches plane (**a**) Ogden model, (**b**) Anssari-Benam model.

Among all the models analyzed, the Yeoh model’s S-shaped pattern in both uniaxial tensile and planar shear stress–strain curves suggests less accuracy in capturing the non-linear hyperelastic behavior of PSAs. The Mooney-Rivlin and Neo-Hookean models, displaying more linear fitting curves, diverge significantly from the experimental findings. Consistent with the findings of previous research by Zhang^18^, it is observed that the Mooney-Rivlin and Neo-Hookean models approximate linear responses and are suitable for describing small to moderate deformations. On the contrary, the third-order Ogden model offers heightened flexibility and affords superior fitting accuracy. The Anssari-Benam model also shows accurate performance, aligning closely with the experimental stress–strain data. While both these two models achieve good fitting results, Anssari-Benam model demonstrates superior convexity preservation compared to the third-order Ogden model, as depicted in the Fig. 7. When the strain energy function loses convexity, the stress and strain are not uniquely related. This can cause problems for the material in numerical simulations.

The comparative analysis led to the selection of the Anssari-Benam model, which provides a more accurate characterization of the non-linear hyperelastic behavior of PSAs. Additionally, this model can achieve the same effectiveness as the third-order Ogden model with fewer parameters. Table 5 shows the Anssari-Benam model parameters obtained through curve fitting based on the uniaxial tensile, compression, and planar shear test results under the different temperature and humidity conditions mentioned above.

**Table 5:** Anssari-Benam model parameter values under varying temperature and humidity conditions.

No.	Temperature (℃)	Humidity (% RH)
1	− 25	0
2	25	0
3	55	0
4	55	90
5	85	0

**Table 6:** Prony coefficients obtained from the generalized Maxwell model.

T (℃)	RH (%)	Prony series	g_i_	k_i_	τ_i_	R^2^
− 25	0	1	0.09987	0.09987	3.5589	0.998
		2	0.29489	0.29489	35.486	
		3	0.26224	0.26224	281.15	
25	0	1	0.10254	0.10254	12.958	0.998
		2	0.56938	0.56938	0.78209	
		3	0.06598	0.06598	79.927	
55	0	1	0.02705	0.02705	855.47	0.998
		2	0.02987	0.02987	58.877	
		3	0.80424	0.80424	3.742	
55	90	1	0.03574	0.03574	42.695	0.995
		2	3.2E-07	3.2E-07	0.61861	
		3	0.8509	0.8509	0.33983	
85	0	1	0.05220	0.05220	1821.2	0.995
		2	0.03002	0.03002	51.686	
		3	0.80997	0.80997	0.51948	

The determination coefficient R^2^ (R-Square) is calculated as follow:

10 $${R}^{2}=1-\frac{\sum_{i}({\widehat{y}}_{i}-{y}_{i}{)}^{2}}{\sum_{i}({\overline{y}}_{i}-{y}_{i}{)}^{2}}$$

where the numerator part represents the sum of the square difference between the real value and the predicted value; the denominator part represents the sum of the square difference between the real value and the mean value. The R-Squared value ranges between 0 and 1 and serves as an indicator of the model's accuracy: a value of 0 implies poor model fitting, indicating that the model fails to accurately predict the data, whereas a value of 1 denotes perfect accuracy, suggesting that the model predicts the data without any error.

### Viscoelastic behavior of PSAs

- Impact of Temperature

**Figure 8:**
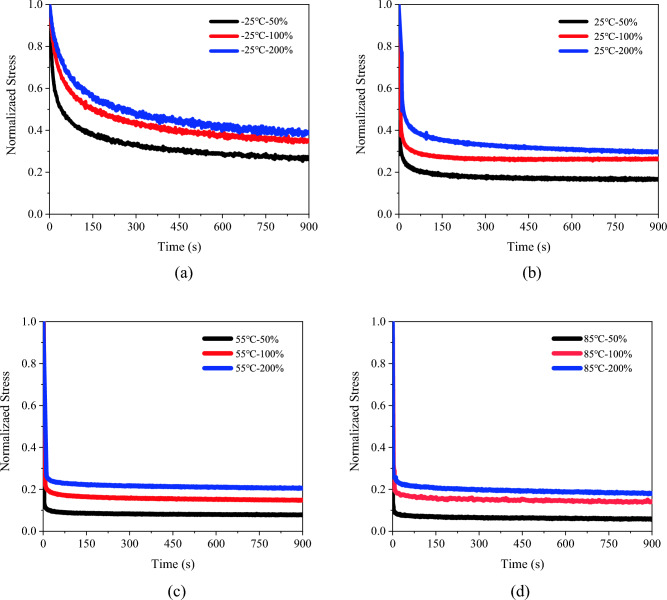
Experimental stress-time curves for PSAs at different temperatures: (**a**) – 25 ℃; (**b**) 25 ℃; (**c**) 55 ℃; (**d**) 85 ℃.

PSAs were tested under different temperature conditions (− 25 °C, 25 °C, 55 °C, and 85 °C) using a uniaxial tensile stress relaxation method. In these tests, PSA samples underwent strain levels of 50%, 100%, and 200%, with three specimens for each experimental group. The resulting stress–strain curves from these tests are depicted in Fig. 8.

The results distinctly show the pronounced effects of both temperature and initial strain on the stress relaxation properties of the PSAs. Notably, at a constant temperature, stress equilibrium values post-relaxation demonstrates an increasing trend with higher initial strain levels. This trend is evident in Fig. 8, where normalized stress at equilibrium is greater for larger initial strains, indicating less stress relaxation at these higher strains—a phenomenon supported by other literature^1^. This behavior is attributable to the viscoelastic nature of PSAs. At elevated initial strains, the internal structure of the adhesive undergoes more significant deformation. This leads to greater alignment or stretching of the polymer chains and adhesive network, resulting in increased resistance to further deformation^25^. During relaxation, the internal structures may not completely revert to their original state, particularly at higher strain levels, likely due to physical entanglements and possible irreversible changes within the polymer network. Consequently, at higher initial strains, PSAs exhibit a larger residual stress, maintaining a higher normalized stress even after relaxation. The reduced relaxation at these strains also reflects the material's non-linear viscoelastic response, which becomes more evident at higher deformation levels.

In terms of temperature impact, the stress relaxation behavior of PSAs also varies markedly. The curves in Fig. 8 reveal that normalized stress decreases as temperature increases. At − 25 °C, the curve shows the highest normalized stress over time, whereas at 85 °C, it is significantly lower. This trend highlights the influence of temperature on the stress relaxation properties of PSAs, in line with their viscoelastic and temperature-sensitive nature. At higher temperatures, the increased thermal energy enhances polymer chain mobility and reduces internal friction, leading to a more rapid relaxation process. This behavior aligns with the time–temperature superposition principle typical of viscoelastic materials, where higher temperatures effectively accelerate relaxation processes^6^. Additionally, the temperature affects the relaxation mechanisms: at lower temperatures, PSAs exhibit a predominantly elastic response due to restricted molecular mobility, while at higher temperatures, a more pronounced viscoelastic response emerges due to increased molecular rearrangement^25^.

**Figure 9:**
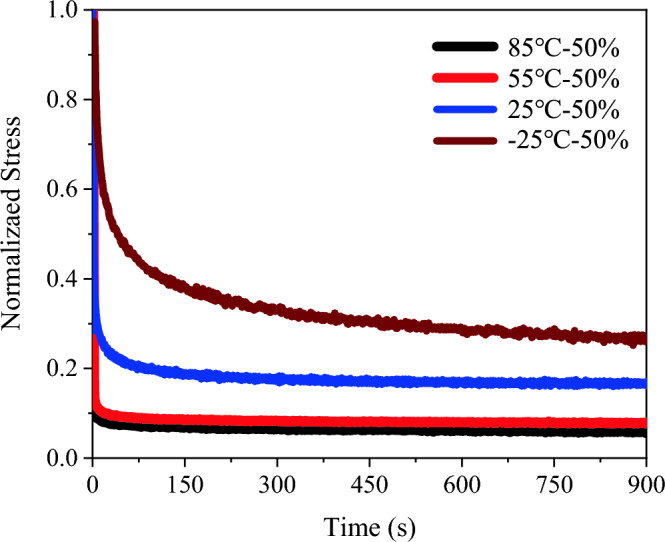
Experimental stress-time curves for PSAs at different temperatures under a constant initial strain of 50%.

Figure 9 shows that as temperature increases, normalized stress in PSAs decreases over time. The curve at − 25 °C shows the highest normalized stress, while at 85 °C, it is significantly lower. This behavior is attributed to the viscoelastic and temperature-sensitive nature of PSAs. Elevated temperatures enhance polymer chain mobility and reduce internal friction, leading to faster stress relaxation. This aligns with the time–temperature superposition principle in viscoelastic materials, where higher temperatures accelerate relaxation processes^26^. Temperature variations also lead to different relaxation mechanisms: at lower temperatures, PSAs show a predominantly elastic response with slower relaxation due to restricted molecular mobility, whereas higher temperatures induce a more pronounced viscoelastic response through facilitated molecular rearrangement^27^.

- Impact of Humidity

**Figure 10:**
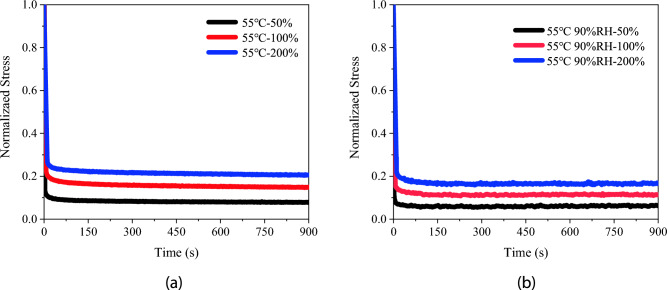
Experimental stress-strain curves for PSAs at varied humidity levels: (**a**) 55 ℃; (**b**) 55 ℃ 90% RH.

**Figure 11:**
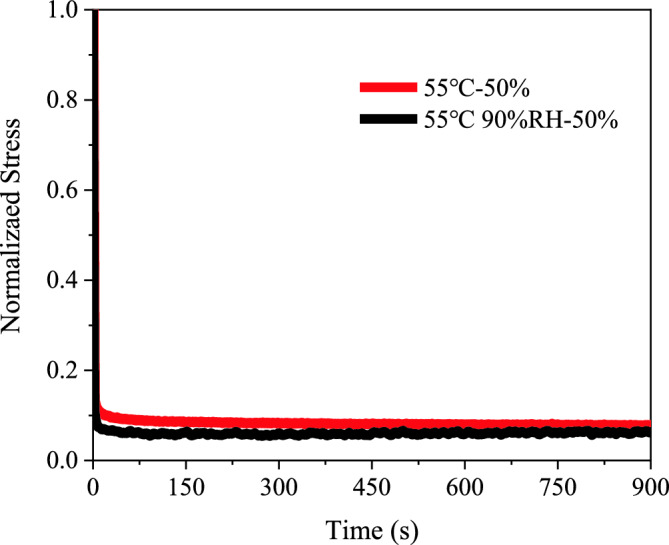
Experimental Stress-Time Curves for PSAs at varied humidity levels with a constant initial strain of 100%.

This section examines the influence of humidity on the viscoelastic properties of PSAs by conducting experiments at a constant temperature of 55 °C under two humidity levels: 0% RH and 90% RH. Using a uniaxial tensile stress relaxation method, PSAs were tested at strain levels of 50%, 100%, and 200%, with three specimens for each condition. The resulting stress–strain curves, shown in Figs. 10 and 11, reveal that at 90% RH, PSAs exhibit lower normalized stress over time compared to those in a dry environment, indicating enhanced stress relaxation due to humidity.

As demonstrated in Figs. 10 and 11, PSAs tested in a higher humidity environment at 90% RH exhibit lower normalized stress levels over time compared to those in a dry environment. This observation suggests an enhancement in the stress relaxation process of PSAs due to humidity^28^. Under these humid conditions, PSAs maintain lower normalized stress for an extended duration, indicating accelerated stress relaxation. Humidity influences the stress relaxation behavior of PSAs through several mechanisms. Firstly, moisture serves as a plasticizer, increasing the mobility of polymer chains and paradoxically leading to a material that is more elastic, thereby accelerating the relaxation rate^29^. Secondly, moisture absorption causes the polymer matrix to swell, expanding its internal structure, which likely increases chain mobility and results in lower stress levels over time^30^. Finally, the presence of humidity facilitates hydrogen bonding between polymer chains and water molecules, which promotes molecular rearrangement and consequently lowers stress levels^22^. These collective effects significantly modulate the viscoelastic response of PSAs in the presence of humidity.

- Constitutive Model Investigation

At present, the widely recognized models for viscoelastic constitutive behavior include the Maxwell, Kelvin, three-parameter, and generalized Maxwell models. The Prony series method is applied to effectively determine these viscoelastic parameters, particularly for the purpose of modeling the stress relaxation behaviors in PSAs^5^.

**Figure 12:**
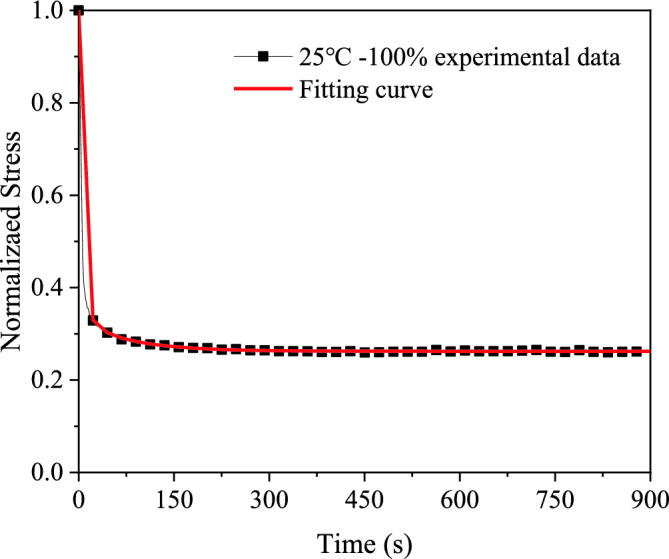
Fitting result of stress relaxation test.

Differing from the fitting approach used for hyperelastic constitutive parameters, the relationship between the relaxation modulus and time can be directly calculated from stress relaxation experiments. Subsequently, the data undergo least-squares fitting to obtain the Prony series of viscoelastic constitutive parameters that meet the required accuracy. Figure 12 displays the experimental fitting curve of PSAs at 25 °C and 100% strain, revealing close alignment between the experimental and fitted data.

Upon calculations, it has been ascertained that a third-order Prony series conforms to the stringent fitting precision requirements. The viscoelastic parameters obtained from the fitting procedure are comprehensively documented in Table 6.

The summary of viscoelastic constitutive parameters and fitting accuracy for PSAs is presented in Table 6. Analysis reveals that the Maxwell model exhibits a robust fit to the experimental data across all testing conditions. The match between the model curves and experimental data is remarkable, as indicated by R^2^ values surpassing 0.99.

## Conclusions

This study provides significant insights into the mechanical behavior of PSAs under various environmental conditions, integral to their application in flexible devices. A key discovery is that the Anssari-Benam model precisely captures the hyperelastic behavior of PSAs, with an R-Square value exceeding 0.97 in all fittings, demonstrating its effectiveness in representing large deformations. Temperature exerts a notable impact on PSA materials, particularly around their T_*g*_. Near the T_*g*_, lower temperatures result in reduced elasticity, while higher temperatures lead to increased stress relaxation due to enhanced polymer chain mobility. In the context of humidity, its presence serves as a plasticizer, rendering PSAs more elastic and reducing stiffness, which is particularly evident in stress relaxation behaviors. These findings underscore the intricate relationship between environmental factors and PSA material properties, emphasizing the need to consider both hyperelastic and viscoelastic responses for their effective use in flexible devices. The study's comprehensive analysis advances our understanding of PSAs in varied operational conditions, providing a solid foundation for their optimized utilization in the evolving field of flexible technology.

### Supplementary Information

Supplementary Information.
